# Proximal tubulopathy in adult sickle cell disease: association of alpha-1 microglobulin

**DOI:** 10.1016/j.clinsp.2026.101064

**Published:** 2026-09-02

**Authors:** Yannick Mompango Engole, Yannick Mayamba Nlandu, Aliocha Natuhoyila Nkodila, Brady Makanzu, Yannick Mvita, Grace Moma Ngoy, Blondy Mvete Mansoni, Raggue Mvibudulu, Beudin Lukoki, Sarah Mukayi Nyemba, Louange Zihirwe Kinja, Ernest Kiswaya Sumaili

**Affiliations:** a Nephrology Unit, Kinshasa University Hospital, University of Kinshasa, Kinshasa, Democratic Republic of the Congo; b Specialized Clinics in Kinshasa, Kinshasa, Democratic Republic of the Congo; c Department of Family Medicine and primary Health care, Protestant University of Congo, Kinshasa, Democratic Republic of the Congo; d Cardiology Unit, Kinshasa University Hospital, Kinshasa, University of Kinshasa, Democratic Republic of the Congo; e Ngiri-Ngiri State Hospital, Kinshasa, Democratic Republic of the Congo; f Mongala Medical Center, Kinshasa, Democratic Republic of the Congo

**Keywords:** Sickle cell disease, Alpha-1 microglobulin, Proximal tubulopathy, Glomerular hyperfiltration

## Abstract

**Background:**

Sickle cell nephropathy remains a major contributor to morbidity in adults with Sickle Cell Disease (SCD), yet proximal tubular injury remains insufficiently characterized, particularly in African settings. Alpha-1 Microglobulin (A1M), a low-molecular-weight protein, may serve as a sensitive biomarker of proximal tubular dysfunction.

**Methods:**

The authors conducted a multicentre observational cross-sectional study among 246 adults with stable SCD in Kinshasa, Democratic Republic of the Congo. Proximal tubulopathy was defined as A1M/Cr ≥ 20 mg/g. Measured Glomerular Filtration Rate (mGFR) was assessed by plasma iohexol clearance. Associations were evaluated using a pre-specified multivariable logistic regression model with multiple imputation for missing data.

**Results:**

Proximal tubulopathy was present in 48% of participants. After adjustment, vaso-occlusive crises during the previous trimester (aOR = 1.96, 95% CI 1.14–3.35), leg ulcers (aOR = 2.11, 95% CI 1.16–3.82), and albuminuria (log-transformed UACR; aOR = 1.53, 95% CI 1.24–1.88) were independently associated with elevated A1M/Cr. Hydroxyurea use was associated with lower odds of proximal tubulopathy (aOR = 0.54, 95% CI 0.31–0.95). In a model adjusted for albuminuria, no independent associations were observed for markers of hemolysis or measured GFR, suggesting that the effects of these factors on tubular injury may be mediated through glomerular injury or are closely intertwined with it. Sensitivity analyses excluding albuminuria from the model (Tables S2–S5) did show associations with leg ulcer, recent VOC, and systolic blood pressure.

**Conclusions:**

Proximal tubular dysfunction is frequent among adults with SCD in this high-burden African setting and was independently associated with markers of disease severity and renal involvement.

## Highlights

• Proximal tubulopathy affects nearly half of adults with SCD.
• A1M is a sensitive biomarker of tubular dysfunction in SCD.
• Disease severity markers strongly associate with tubular injury.
• Hydroxyurea use is linked to lower odds of tubulopathy.
• Tubular and glomerular injuries frequently coexist in SCD.

## Introduction

Sickle Cell Disease (SCD) is an inherited hemoglobin disorder characterized by chronic hemolysis, recurrent vaso-occlusive crises, and progressive multi-organ damage. As life expectancy has improved, Sickle Cell Nephropathy (SCN) has emerged as a major cause of morbidity and mortality among adults with SCD.^1^ Renal involvement in SCD is multifactorial, encompassing glomerular hyperfiltration, albuminuria, and a gradual decline in Glomerular Filtration Rate (GFR), but also tubular injury, which has been described even in patients without overt glomerular damage.^2^

The renal medulla and proximal tubules are particularly vulnerable to ischemia and oxidative stress induced by repeated sickling and chronic hemolysis. Experimental and clinical studies have shown that tubular dysfunction manifested by phosphaturia, glycosuria, and low molecular weight proteinuria can occur early in the course of the disease, even in patients with preserved GFR.^3^, ^4^, ^5^, ^6^ Chronic intravascular hemolysis leads to the release of free hemoglobin and heme, which promote oxidative stress, inflammation, and direct cytotoxicity within tubular epithelial cells.^7^

Among emerging biomarkers, Alpha-1 Microglobulin (A1M), a low-molecular-weight heme-binding glycoprotein that is freely filtered by the glomerulus and almost completely reabsorbed in the proximal tubule, has gained attention as a sensitive indicator of proximal tubular dysfunction.^8^, ^9^, ^10^ In patients with SCD, elevated urinary A1M has been associated with hemolysis-related tubular stress and adverse clinical outcomes, including increased mortality, highlighting its potential value as a marker of tubular dysfunction in sickle cell-related nephropathy.^7^^,^^11^^,^^12^

Despite these observations, evidence regarding proximal tubular injury and A1M in adult African populations with SCD remains limited. Most available studies have been conducted in pediatric cohorts or in high-income settings, often relying on estimated GFR rather than direct measurement, and may not fully capture the renal phenotype observed in adults living in sub-Saharan Africa. This region bears the highest global burden of SCD and is characterized by unique genetic modifiers with the Bantu or Central African phenotype.^13^ environmental exposures, and healthcare constraints, which may influence the expression and progression of renal complications.

Therefore, this study aimed to evaluate urinary A1M as a biomarker of proximal tubular dysfunction in adults with SCD in an African setting and to identify the associated factors with tubular injury.

## Methods

### Study design and participants

This was a multicenter cross-sectional observational study with convenience sampling that was conducted between March 2023 and April 2024 among adults with stable Sickle Cell Disease (SCD) in Kinshasa, Democratic Republic of Congo (DRC). Participants were recruited in collaboration with their primary healthcare providers acrossc 14 centers, including the *Centre For mixed medical for anemia SS, Makala General Referral Hospital*, Bokoko Medical Centre, Akram Hospital, Centre Messie, Clinique Élisabeth, Fondation Amza, Rezodrepa, Codek, Colombe, Kalaki Foundation, and Lisungi.

Eligible participants were adults with confirmed SCD in a steady state. Exclusion criteria included hypertension, diabetes, obesity, urinary tract infection, menstruation, pregnancy, recent vaso-occlusive crisis, fever, acute chest syndrome, or recent (< 1-month) use of Non-Steroidal Anti-Inflammatory Drugs (NSAIDs) or medications interfering with creatinine secretion (e.g., cimetidine, trimethoprim).

The study was approved by the Ethics Committee of the Faculty of Public Health, University of Kinshasa (Approval n° ESP/CE/143/2022). Written informed consent was obtained from all participants prior to inclusion. “This study was reported in accordance with the STROBE (Strengthening the Reporting of Observational Studies in Epidemiology) guidelines.”

### Clinical and laboratory assessments

Demographic and clinical data, including age, sex, body weight, height, and Body Mass Index (BMI), were recorded. Laboratory analyses included:

• Serum cystatin C, C-Reactive Protein (CRP), and ferritin measured by immunonephelometry (I-Chroma 2, Seoul, South Korea);
• Serum creatinine measured by the enzymatic method (Cobas, Roche Diagnostics, Germany);
• Complete blood count determined by impedance (Zybio, Seoul, South Korea);
• Lactate Dehydrogenase (LDH), Aspartate Aminotransferase (AST), and bilirubin (total and direct) determined by kinetic methods on a semi-automated analyzer.

Quantification of hemoglobin S was performed by cellulose acetate electrophoresis using the Gazelle device (Hemex Health, Portland, USA).

The estimated Glomerular Filtration Rate (eGFR) was calculated using the CKD-EPI_crea+cys2021_ equation. The measured GFR (mGFR) was determined by plasma iohexol clearance (Omnipaque 350 mg/mL; GE Healthcare, Belgium). After intravenous injection of 3.45 mL iohexol, 5 mL blood samples were collected at 120-, 180-, 240-, and 300-minutes from the contralateral arm. After centrifugation, samples were stored at −80 °C and shipped to the University of Liège (Belgium) for analysis. Plasma iohexol concentrations were measured by Liquid Chromatography tandem Mass Spectrometry (LC-MS/MS) in an ISO 15,189-accredited laboratory participating in external quality assessment (Equalis AB, Sweden).^14^^,^^15^ Measured GFR was derived from iohexol plasma clearance using the slope-intercept method with Brøchner-Mortensen correction.^16^^,^^17^ and was indexed to body surface area calculated according to the Du Bois and Du Bois equation.^18^

Urine samples were collected from the first morning void. The urinary Albumin-to-Creatinine Ratio (UACR) was calculated as albumin (mg/L) divided by creatinine (mg/L), both determined by immunoturbidimetry (Cobas, Roche Diagnostics, Germany). The α1-Microglobulin to Creatinine ratio (A1M/Cr) was measured using the same analyzer and expressed in mg/g.

Transthoracic Doppler echocardiography was performed by a single experienced cardiologist using a Mindray Consona 8 ultrasound system (Mindray, Shenzhen, China). During diastole, the left ventricular end-diastolic diameter, interventricular septal thickness, and posterior wall thickness were measured. Left Ventricular Mass (LVM) was calculated and indexed to Body Surface Area (BSA). The Left Ventricular Ejection Fraction (LVEF) was determined using the modified biplane Simpson’s method, while the left atrial volume was also indexed to BSA. Transmitral flow velocities (E and A waves) and the E/A and E/E′ ratios were obtained using pulsed-wave Doppler.

### Operational definitions

• The primary outcome of the study was proximal tubulopathy, defined as an A1M/Cr ≥ 20 mg/g [9]
• Glomerular hyperfiltration: mGFR ≥ 135 mL/’/1.73 m^2^.^19^
• Stable SCD: Defined as the absence of vaso-occlusive crises, fever, or any acute complication for more than one month.^20^

### Statistical analysis

Data were compiled into a Microsoft Excel 2010 database from patients followed for sickle cell disease, and statistical analyses were performed using IBM SPSS for Windows, version 25. Sociodemographic, clinical, and biological characteristics of participants were compared according to proximal tubulopathy status (proximal tubulopathy vs. without proximal tubulopathy).

Continuous variables were expressed as mean ± Standard Deviation (SD) or as median with Interquartile Range (IQR), depending on data distribution. Normality of distributions was assessed using the Shapiro-Wilk test. For normally distributed variables, comparisons between groups were performed using Student’s *t*-test, whereas the Mann-Whitney *U test* was used for non-normally distributed variables. Categorical variables were presented as absolute numbers and percentages. Group comparisons were performed using Pearson’s Chi-Square test, and Fisher’s exact test was applied when expected cell counts were small.

A multivariable logistic regression model was pre-specified to identify factors associated with proximal tubulopathy. Covariates were selected a priori based on clinical relevance and existing literature, and included age, sex, hydroxyurea use, vaso-occlusive crises in the previous trimester, leg ulcers, lactate dehydrogenase, fetal hemoglobin level, C-reactive protein, measured glomerular filtration rate, and urinary albumin-to-creatinine ratio. No stepwise variable selection procedure was used. A p-value < 0.05 was considered statistically significant.

In the analytic cohort with both available A1M/Cr and measured GFR (*n* = 246), no missing data were observed for A1M/Cr, UACR, or measured GFR. Missingness was low (< 4%) for most clinical and laboratory covariates, except for fetal hemoglobin (17.9%). Because fetal Hb and selected clinical variables had incomplete observations, missing data were handled using multiple imputation by chained equations (20 imputations). The imputation model included all covariates and the outcome variable to ensure model congeniality. Estimates were pooled using Rubin’s rules. Complete-case sensitivity analyses yielded similar effect estimates. The extent of missing data for all variables included in the analytic cohort is detailed in Supplementary Table S1.

## Results

### Study population and prevalence of proximal tubulopathy

A total of 246 adults with sickle cell disease were included in the analysis. Among them, 118 patients (48.0%) met the criteria for proximal tubulopathy, while 128 (52.0%) had no evidence of proximal tubular involvement.

### General characteristics according to proximal tubulopathy status

Table 1 summarizes the demographic and clinical characteristics of the study population according to urinary α1-microglobulin status. Participants with elevated A1M/Cr more frequently reported vaso-occlusive crises during the previous trimester and leg ulcers, and were less likely to be receiving hydroxyurea. Other clinical manifestations and lifestyle factors were similarly distributed between groups.

**Table 1:** General characteristics of population studied.

Variable	Overall (*n* = 246)	A1M < 20 mg/g (*n* = 128)	A1M ≥ 20 mg/g (*n* = 118)	p
Sex				0.452
Female	146 (59.3)	75 (58.6)	71 (60.2)	
Male	100 (40.7)	53 (41.4)	47 (39.8)	
Folic acid use	226 (91.9)	117 (91.4)	109 (92.4)	0.484
Hydroxyurea use	88 (35.8)	52 (40.6)	36 (30.5)	**0.027**
Surgery	57 (23.2)	27 (21.1)	30 (25.4)	0.257
VOC in last trimester	105 (42.7)	42 (32.8)	63 (53.4)	**0.001**
FH-SCD	142 (57.7)	73 (57.0)	69 (58.5)	0.460
History of Blood transfusion	222 (90.2)	112 (87.5)	110 (93.2)	0.097
Leg ulcer	64 (26.0)	24 (18.8)	40 (33.9)	**0.005**
Priapism	17 (6.9)	9 (7.0)	8 (6.8)	0.570
Retinopathy	38 (15.4)	23 (18.0)	15 (12.7)	0.168
Aseptic necrosis of femoral head	44 (17.9)	24 (18.8)	20 (16.9)	0.421
Osteomyelitis	3 (1.2)	1 (0.8)	2 (1.7)	0.469
Stroke	3 (1.2)	1 (0.8)	2 (1.7)	0.469
Tabacco	23 (9.3)	9 (7.0)	14 (11.9)	**0.046**
Alcohol	60 (24.4)	32 (25.0)	28 (23.7)	0.467
Glucosuria	116 (47.2)	64 (50.0)	52 (44.1)	0.211
Fetal Hb > 15	36 (16.4)	13 (11.7)	23 (21.3)	0.083

Data are expressed as mean ± standard deviation, absolute (n) and relative (in percent) frequencies.

Fetal Hb, Fetal Haemoglobin; VOC, Vaso-Occlusive Crisis; FH-SCD, Familial History of Sickle Cell Disease.

As shown in Table 2, most hemodynamic and echocardiographic parameters were comparable across A1M/Cr categories. Minor differences were observed for respiratory rate and cardiac output; however, these variables were further evaluated in multivariable analyses to account for potential confounding.

**Table 2:** Hemodynamic and echocardiographic parameters of population studied.

Variable	Overall (*n* = 246)	A1M < 20 mg/g (*n* = 128)	A1M ≥ 20 mg/g (*n* = 118)	p
Age	26.0 ± 7.8	25.4 ± 7.7	26.6 ± 8.0	0.203
SBP	104.8 ± 13.0	105.6 ± 12.1	103.9 ± 13.9	0.344
DBP	59.2 ± 9.8	59.7 ± 9.0	58.7 ± 10.6	0.449
HR	84.9 ± 12.1	84.5 ± 12.4	85.2 ± 11.8	0.675
RR	21.4 ± 2.9	21.0 ± 2.7	21.8 ± 3.0	**0.035**
Weight	49.9 ± 8.7	50.5 ± 8.2	49.2 ± 9.2	0.269
Height	160.0 ± 17.2	159.0 ± 22.7	161.0 ± 8.2	0.374
BMI	19.2 ± 2.9	19.4 ± 2.5	19.0 ± 3.3	0.375
PWV	5.0 (4.4–5.7)	5.0 (4.4–6.1)	5.0 (4.5–5.6)	0.788
SVR	916.8 (681.5‒1137.8)	932.0 (678.5–1243.2)	901.5 (689.2–1115.0)	0.627
LVEF	68.0 ± 9.6	66.5 ± 10.4	69.4 ± 8.7	0.128
LVEDD	50.3 ± 5.7	50.3 ± 6.0	50.2 ± 5.5	0.961
RVEDD	28.2 ± 5.0	28.4 ± 5.4	28.0 ± 4.4	0.689
ILVM	109.6 ± 27.1	108.0 ± 29.1	111.3 ± 25.0	0.551
IVS	8.8 (7.7–9.8)	8.5 (7.7–9.8)	8.8 (8.1–10.0)	0.174
PW	9.2 (8.4–10.3)	9.1 (8.3–10.1)	9.4 (8.4–10.3)	0.404
IVC	12.65 (10.15–14.55)	12.5 (9.9–14.2)	12.7 (10.4–14.7)	0.495
E/A	1.51 (1.22–1.72)	1.5 (1.2–1.8)	1.5 (1.2–1.7)	0.547
E/E'	7.68 (6.44–8.93)	7.6 (6.3–8.9)	7.7 (6.4–9.3)	0.754
TRV	1.67 (1.45–1.83)	1.66 (1.45–1.82)	1.68 (1.45–1.87)	0.590
TAPSE	2.7 (2.3–2.9)	2.6 (2.4–3.1)	2.7 (2.3–2.9)	0.824
Cardiac output	6.71 (5.34–8.37)	6.61 (5.32–8.11)	6.75 (5.35–8.55)	0.550

Data are expressed as mean ± standard deviation, median (interquartile range), absolute (n) frequencies.

BMI, Body Mass Index; DBP, Diastolic Blood Pressure; HR, Heart Rate; IVC, Inferior Venous Cave; IVS, Interventricular Septum; LVEDD, Left Ventricular End Diastolic Diameter; LVEF, Left Ventricular Ejection Fraction; iLVM, Indexed Left Ventricular Mass; PW, Posterior Wall; PWV, Pulse Wave Velocity; RVEDD, Right Ventricular End Diastolic Diameter; TAPSE, Tricuspid Annular Plane Systolic Excursion; SBP, Systolic Blood Pressure; SVR, Systemic Vascular Resistance.

Table 3 presents renal and biological parameters stratified by A1M/Cr status for descriptive purposes. As expected, urinary α1-microglobulin and albumin-to-creatinine ratio were higher in participants classified with proximal tubulopathy, reflecting the definition of the outcome. Differences were also observed for markers of haemolysis and inflammation, as well as measured GFR, which were subsequently examined in multivariable models.

**Table 3:** Biological parameters of population studied.

Variable	Overall (*n* = 246)	A1M < 20 mg/g (*n* = 128)	A1M ≥ 20 mg/g (*n* = 118)	p
A1M/creat, mg/g	18.9 (9.2–42.5)	9.9 (5.5–14.0)	43.9 (30.1–70.8)	**<0.001**
UACR, mg/g	10.9 (4.1–38.8)	7.2 (3.3–16.0)	19.9(8.0–84.9)	**<0.001**
Creatinine, mg/dL	0.58 (0.49–0.70)	0.6 (0.5–0.7)	0.6 (0.5–0.7)	0.773
Cystatin C, mg/L	0.86 (0.75–0.99)	0.8 (0.7–0.9)	0.9 (0.8–1.1)	**<0.001**
mGFR (mL/min/1.73m^2^)	126 (110–136)	128 (105–151)	115 (96–128)	**<0.001**
CKD-EPI_crea-cys_ (mL/min/1.73m^2^)	123 (103–146)	130 (103–152)	119 (103–132)	0.503
Urea, mmoL/L	4.9 (3.3–6.0)	5.0 (3.8–6.1)	4.7 (3.3–5.9)	0.499
Hb, g/dL	7.5 ± 1.7	7.5 ± 1.7	7.4 ± 1.8	0.651
Hct, %	23.5 ± 5.6	23.7 ± 5.5	23.3 ± 5.7	0.600
RBC, × 10^6^/mm^3^	2.7 ± 0.7	2.8 ± 0.8	2.7 ± 0.7	0.212
MCV, fl	87.6 ± 10.6	87.4 ± 10.0	87.8 ± 11.2	0.781
MCH, %	27.8 ± 4.1	27.8 ± 4.1	27.9 ± 4.1	0.822
MCHC, %	31.4 ± 2.6	31.2 ± 3.3	31.6 ± 1.5	0.236
Reticulocyte count, %	16.5 ± 2.0	16.4 ± 2.0	16.6 ± 2.0	0.596
WBC, × 10^3^/mm^3^	10.4 (8.8–13.8)	10.9 (8.6–14.1)	10.4 (9.0–13.3)	0.781
Neutrophils, %	48.9 (40.7–58.6)	48.6 (39.4–56.5)	50.0 (41.0–60.7)	0.355
Lymphocytes, %	39.1 (30.6–46.5)	39.5 (32.0–46.2)	38.7 (28.2–46.9)	0.531
Monocytes, %	7.7 (5.8–10.7)	7.7 (6.0–10.7)	7.7 (5.7–10.5)	0.620
Eosinophils, %	2.3 (1.2–4.1)	2.0 (1.1–3.8)	2.4 (1.3–4.8)	0.301
Basophils, %	0.3 (0.2–0.4)	0.2 (0.2–0.4)	0.3 (0.2–0.4)	0.358
Platelets, × 10^3^/mm^3^	156.2 (150.6–162.9)	156.2 (150.7–162.9)	156.7 (150.4–163.2)	0.735
AST, IU/L	33.0 (27.0–44.6)	32.4 (27.0–44.0)	44.1 (27.0–54.9)	**0.011**
Total Bili, mg/L	0.99 (0.91–1.20)	1.0 (0.9–1.2)	1.0 (0.9–1.1)	0.278
Direct Bili, mg/L	0.30 (0.24–0.51)	0.3 (0.2–0.6)	0.3 (0.2–0.4)	0.437
LDH, IU/L	210.0 (192.9–264.9)	202.0 (191.0–264.0)	299.7 (193.2–383.3)	**0.006**
Ferritin, ng/mL	267.1 (96.5–668.9)	254.8 (94.7–656.7)	279.5 (95.7–701.0)	0.890
CRP, mg/L	8.12 (3.24–18.18)	7.2 (2.9–18.2)	9.5 (4.2–16.6)	**0.032**
Fetal Hb, %	9.0 (8.0–13.0)	9.0 (8.0–12.0)	9.0 (8.0–14.0)	0.659

Data are expressed as mean ± standard deviation, median (interquartile range), absolute (n) and relative (in per cent) frequencies.

A1M/crea, α1-Micrglobulin to Creatininuria; AST, Aspartate Aminotransferase; CKD-EPIcrea+cys, Chronic Kidney Disease-Epidemiology combining creatinine and cystatine; CRP, C-Reactive Protein; eGFR, estimated Glomerular Filtration Rate; Hb, Haemoglobin; Hct, Haematocrit; LDH, Lactate Dehydrogenase; MCV, Mean Corpuscular Volume; MCH, Mean Corpuscular Hemoglobin; MCHC, Mean Corpuscular Hemoglobin Concentration; RBC, Red Blood Cells; UACR, Urinary Albumin to Creatinine Ratio; WBC, White Blood Cells.

Table 4 presents the results of a pre-specified multivariable logistic regression model with elevated A1M/Cr (≥ 20 mg/g) as the dependent variable in the entire cohort (*n* = 246). Hydroxyurea use was independently associated with lower odds of elevated A1M/Cr (aOR = 0.54, 95% CI 0.31–0.95), whereas vaso-occlusive crises during the previous trimester (aOR = 1.96, 95% CI 1.14–3.35), leg ulcers (aOR = 2.11, 95% CI 1.16–3.82), and log-transformed UACR (aOR = 1.53, 95% CI 1.24–1.88) were independently associated with higher odds of elevated A1M/Cr. Additional sensitivity analyses excluding UACR and stratified according to albuminuria category (A1 vs. A2/A3) are presented in the Supplementary Material (Tables S2–S5) and yielded consistent findings, with clinical severity markers such as vaso-occlusive crises and leg ulcers remaining the most robust predictors.

**Table 4:** Associated factors of proximal tubulopathy.

Variable	aOR (95% CI)	p
Age (per 5-year increase)	1.11 (0.94–1.32)	0.22
Female sex	0.78 (0.46–1.33)	0.36
Hydroxyurea use	0.54 (0.31–0.95)	**0.031**
VOC in previous trimester	1.96 (1.14–3.35)	**0.014**
Leg ulcer	2.11 (1.16–3.82)	**0.014**
History of blood transfusion	1.80 (0.60–5.38)	0.30
Hemoglobin (per 1 *g*/dL)	0.99 (0.82–1.19)	0.91
Reticulocyte (per 1%)	1.02 (0.87–1.20)	0.82
LDH (per 100 IU/L)	1.03 (0.85–1.25)	0.74
HbF (per 5% increase)	1.14 (0.87–1.50)	0.35
mGFR (per 10 mL/min/1.73m²)	0.98 (0.91–1.07)	0.69
UACR (log-transformed)	1.53 (1.24–1.88)	**<0.001**

## Discussion

In this multicentre cross-sectional study of adults with sickle cell disease in Kinshasa, proximal tubulopathy was frequent, affecting nearly half of participants. This prevalence is within the range reported in previous studies (approximately 40%–70%), although comparisons are limited by heterogeneity in definitions and biomarkers used to characterize proximal tubular dysfunction.^21^^,^^22^ Several studies have described early tubular abnormalities, including increased urinary excretion of low-molecular weight proteins, glycosuria, phosphaturia and altered urate handling, often in patients with preserved glomerular filtration rate.^23^^,^^24^ Variability across studies likely reflects differences in diagnostic criteria and biomarkers used to define proximal tubular dysfunction. Beyond elevated urinary α1-microglobulin, early abnormalities such as normoglycaemic glycosuria, phosphaturia, and increased fractional excretion of uric acid have been described, often in patients with preserved glomerular filtration rate. Globally, studies from Europe and Although proximal tubulopathy has not always been explicitly defined, several studies have demonstrated increased urinary excretion of low molecular weight proteins (such as β2-microglobulin or retinol-binding protein), supporting the presence of proximal tubular dysfunction.^25^^,^^26^ In this context, the prevalence observed in the present study is consistent with global data suggesting that tubular injury is common in SCD and frequently coexists with glomerular abnormalities.

Elevated urinary α1-Microglobulin-to-Creatinine ratio (A1M/Cr) was independently associated with markers of disease severity and renal involvement. In a pre-specified multivariable logistic regression model with multiple imputation, recent vaso-occlusive crises, leg ulcers, and albuminuria were independently associated with elevated A1M/Cr, whereas hydroxyurea use was associated with lower odds of elevated A1M/Cr.

The association between recent vaso-occlusive crises and elevated A1M/Cr indicates that proximal tubular dysfunction clusters with recent clinical disease activity. Although causality cannot be inferred, this finding is consistent with the known susceptibility of the renal microcirculation, particularly the medulla and proximal tubules, to hypoxic and vaso-occlusive stress in SCD.^27^, ^28^, ^29^ Similar associations between disease activity, ischemic burden, and renal injury have been reported previously.^30^

Leg ulcers, a recognized marker of chronic vasculopathy and endothelial dysfunction in SCD, were also independently associated with elevated A1M/Cr. This observation aligns with prior reports linking leg ulcers to more severe systemic disease and increased risk of end-organ damage, including renal involvement.^31^, ^32^, ^33^ suggesting that chronic microvascular dysfunction may represent a shared underlying feature.

Albuminuria showed a strong and independent association with elevated A1M/Cr after adjustment, supporting the concept that glomerular and tubular abnormalities frequently coexist in adult SCD and may reflect parallel pathways of kidney injury. Albuminuria is a well-established early manifestation of sickle cell nephropathy and is associated with progression to chronic kidney disease. Multiple mechanisms may impair megalin-cubilin function, including chronic hemolysis, excess free hemoglobin, oxidative stress, medullary ischemia, hyperfiltration, metabolic acidosis, and tubular overload. The high prevalence of mixed phenotypes further reflects impaired reabsorption of filtered albumin through the megalin-cubilin receptor complex in the proximal tubule.^30^^,^^34^^,^^35^ These findings extend these observations by demonstrating a close association between albuminuria and tubular protein excretion even among patients with preserved measured GFR. Because UACR is strongly correlated with A1M/Cr, inclusion of albuminuria in the fully adjusted model may attenuate associations between upstream biological factors such as hemolysis or hyperfiltration and tubular proteinuria. This statistical phenomenon likely reflects shared or mediated pathways linking glomerular and tubular injury in sickle cell nephropathy rather than the absence of a biological contribution of hemolysis-related mechanisms. Therefore, the absence of independent associations in this model should not be interpreted as evidence against a biological role of hemolysis or hyperfiltration, but rather as indicating no additional independent contribution beyond albuminuria within this model specification.

Hydroxyurea use was independently associated with lower odds of elevated A1M/Cr. However, given the observational and cross-sectional design of this study, this association should not be interpreted as causal. Hydroxyurea use may reflect differences in disease management, healthcare access, or treatment adherence that could confound this relationship. While no protective effect can be inferred from this cross-sectional analysis, this association is consistent with previous observational studies reporting lower prevalence of albuminuria and slower renal function decline among hydroxyurea-treated patients.^36^, ^37^, ^38^

Markers of hemolysis, including lactate dehydrogenase and reticulocyte count, as well as fetal hemoglobin levels, were not independently associated with elevated A1M/Cr after full adjustment, suggesting that their relationship with tubular dysfunction may be mediated through albuminuria or disease severity and cannot be disentangled in a cross-sectional framework.^33^^,^^39^

Overall, these findings suggest that tubular injury represents an important component of sickle cell nephropathy. Longitudinal studies are required to determine temporal relationships and prognostic implications.

### Strengths of the study

This study has several important strengths. First, it focuses on proximal tubular dysfunction, an underexplored aspect of sickle cell nephropathy, particularly in adult African populations. Second, the use of urinary α1-microglobulin provides a sensitive and biologically plausible marker of proximal tubular injury. Third, measured GFR using plasma iohexol clearance was available in a large proportion of participants, allowing a more accurate assessment of renal function than reliance on estimated GFR alone. Fourth, the use of a pre-specified multivariable model with multiple imputation strengthens the robustness of the findings and addresses key methodological concerns related to missing data and overfitting.

### Limitations

Several limitations should be acknowledged. The cross-sectional design precludes any inference regarding causality or temporal relationships between disease severity, tubular dysfunction, and renal outcomes. The definition of proximal tubulopathy based on an A1M/Cr threshold ≥ 20 mg/g, although supported by prior studies, has not been universally validated across different populations, particularly in African settings, and may limit the generalizability of the present findings. Only a single tubular biomarker was used, which may not fully capture the spectrum of proximal tubular abnormalities. Residual confounding cannot be excluded despite comprehensive multivariable adjustment. The study relied on convenience sampling within a single urban region, which may limit generalizability to other SCD populations. Additionally, detailed information on treatment duration, dosing, and adherence was not systematically available in this cohort. Finally, data on genetic modifiers such as α-thalassemia status or APOL1 variants were not available and could not be accounted for.

## Conclusion

In conclusion, proximal tubular dysfunction, as reflected by elevated urinary α1-microglobulin, is frequent among adults with sickle cell disease in this high-burden African setting and is independently associated with markers of disease severity and renal involvement. These findings suggest that tubular injury represents an important component of sickle cell nephropathy that may not be detected by conventional measures of kidney function. Prospective longitudinal studies are needed to clarify the prognostic significance of A1M and to determine whether interventions such as hydroxyurea can modify the trajectory of tubular injury and renal outcomes in sickle cell disease.

## Authors’ contributions

All authors of the manuscript participated in conducting and preparing the study.

Yannick Engole: Conceptualization; data curation; formal analysis; funding acquisition; investigation; methodology; project administration; supervision; visualization; writing-original draft; writing-review and editing.

Yannick Nlandu: Data curation; formal analysis; writing-original draft; writing-review and editing.

Aliocha Nkodila: Data curation; formal analysis; software; writing-original draft; writing-review and editing.

Brady Makanzu: Investigation; resources; writing-review and editing.

Yannick Mvita: Investigation; validation; writing-review and editing.

Grace Ngoy: Investigation; ressources; writing-review and editing.

Blondy Mansoni: Resources; writing-review and editing.

Raggue Mvibudulu: Ressources; writing-review and editing.

Beudin Lukoki: Investigation; writing-review and editing.

Sarah Nyemba: Funding acquisition; investigation; resources; writing-review and editing.

Louange Kinja: Investigation; resources; writing-review and editing.

Ernest Sumaili: Conceptualization; funding acquisition; methodology; project administration; supervision; validation; writing-original draft; writing-review and editing.

## Funding

This research did not receive any specific funding from public, commercial, or not-for-profit organizations.

Supplementary Material.

Supplementary Tables S1–S5 are available online.

## Declaration of competing interest

The authors declare no conflicts of interest.

## Data Availability

In order to protect the privacy of study participants, as agreed with them in the informed consent form, the data cannot be shared openly. However, it can be made available by email to anyone who writes to us and wishes to preserve the confidentiality of this information.

## Appendix Supplementary materials

Image, application 1

Image, application 2

## Footnotes

Supplementary material associated with this article can be found, in the online version, at doi:10.1016/j.clinsp.2026.101064.
