# Supplementary material for: Proximal tubulopathy in adult sickle cell disease: association of alpha-1 microglobulin
Source: Clinics (Sao Paulo). 2026 Sep 2;81:101064. doi: 10.1016/j.clinsp.2026.101064 (PMC13571299; doi:10.1016/j.clinsp.2026.101064)
Supplement: Supplementary file 2 [file mmc2.docx]

**Supplementary Table 1. Missing data in the analytic cohort with available A1M (N = 246)**

| **Variables** | **Missing (n)** | **Total (n)** | **Missing (%)** |
| --- | --- | --- | --- |
| Age | 0 | 246 | 0 |
| Sex | 0 | 246 | 0 |
| Hydroxyurea use | 8 | 246 | 3.25 |
| VOC in previous trimester | 8 | 246 | 3.25 |
| Leg ulcer | 8 | 246 | 3.25 |
| History of blood transfusion | 8 | 246 | 3.25 |
| Hemoglobin | 0 | 246 | 0 |
| Reticulocyte count | 1 | 246 | 0.4 |
| LDH | 4 | 246 | 1.62 |
| Fetal Hb | 44 | 246 | 17.9 |
| UACR | 0 | 246 | 0 |
| Measured GFR | 0 | 246 | 0 |

Abbreviations: GFR: Glomerular filtration rate; LDH: Lactico dehydrogenase; Hb: Haemoglobin; UACR: Urinary albumin to creatinine ratio; VOC: Vaso-occlusive crisis

**2. Additional sensitivity analyses (excluding UACR and stratified by albuminuria status).**

**Table S2. Model A (with UACR)**

| **Variable** | **Beta** | **SE** | **OR (expβ)** | **95% CI (OR)** | **p-value** |
| --- | --- | --- | --- | --- | --- |
| Ln (UACR) | 0.43 | 0.05 | 1.537 | 1.394; 1.696 | **<0.001** |
| LDH (per 100 IU/L) | 0.02 | 0.06 | 1.020 | 0.907; 1.148 | 0.7 |
| mGFR (per 10 mL/min/1.73m²) | -0.04 | 0.05 | 0.961 | 0.871; 1.060 | 0.41 |
| Age (per 10 years) | 0.06 | 0.04 | 1.062 | 0.982; 1.148 | 0.15 |
| Male sex | 0.11 | 0.09 | 1.116 | 0.936; 1.332 | 0.23 |
| Leg Ulcer | 1.141 | 0.414 | 3.130 | 1.390; 7.046 | **0.005** |
| SBP (per 10 mmHg) | -0.08 | 0.05 | 0.923 | 0.837; 1.018 | 0.12 |
| Hemoglobin (per 1 g/dL) | -0.05 | 0.04 | 0.951 | 0.880; 1.029 | 0.22 |
| Hydroxyurea use | -0.09 | 0.1 | 0.914 | 0.751; 1.112 | 0.34 |
| Recent VOC | 0.37 | 0.13 | 1.448 | 1.122; 1.868 | **0.004** |

**Table S3. Model B (without UACR)**

| **Variable** | **Beta** | **SE** | **OR (expβ)** | **95% CI (OR)** | **p-value** |
| --- | --- | --- | --- | --- | --- |
| LDH (per 100 IU/L) | 0.03 | 0.06 | 1.030 | 0.916; 1.159 | 0.62 |
| mGFR (per 10 mL/min/1.73m²) | -0.03 | 0.05 | 0.970 | 0.880; 1.070 | 0.53 |
| Age (per 10 years) | 0.07 | 0.04 | 1.073 | 0.992; 1.160 | 0.09 |
| Male sex | 0.14 | 0.09 | 1.150 | 0.964; 1.372 | 0.12 |
| SBP (per 10 mmHg) | -0.12 | 0.06 | 0.887 | 0.789; 0.998 | 0.068 |
| Hemoglobin (per 1 g/dL) | -0.06 | 0.04 | 0.942 | 0.871; 1.019 | 0.14 |
| Hydroxyurea use | -0.1 | 0.1 | 0.905 | 0.744; 1.101 | 0.29 |
| Recent VOC | 0.74 | 0.353 | 2.096 | 1.049; 4.187 | **0.035** |
| Leg Ulcer | 1.105 | 0.39 | 3.019 | 1.406; 6.484 | **0.048** |

**Table S4. Model C: With Albuminuria A1**

| **Variable** | **Beta** | **SE** | **OR (expβ)** | **95% CI (OR)** | **p-value** |
| --- | --- | --- | --- | --- | --- |
| LDH | 0.02 | 0.05 | 1.020 | 0.925; 1.125 | 0.68 |
| mGFR | -0.01 | 0.04 | 0.990 | 0.915; 1.071 | 0.95 |
| SBP | -0.13 | 0.06 | 0.878 | 0.781; 0.988 | **0.046** |
| Recent VOC | 0.29 | 0.15 | 1.336 | 0.996; 1.793 | 0.051 |
|  |  |  |  |  |  |

**Table S5. Model C: With Albuminuria A2, A3**

| **Variable** | **Beta** | **SE** | **OR (expβ)** | **95% CI (OR)** | **p-value** |
| --- | --- | --- | --- | --- | --- |
| LDH | 0.05 | 0.12 | 1.051 | 0.831; 1.330 | 0.68 |
| mGFR | -0.07 | 0.17 | 0.932 | 0.668; 1.301 | 0.68 |
| SBP | -0.04 | 0.11 | 0.961 | 0.774; 1.192 | 0.73 |
| Recent VOC | 0.67 | 0.29 | 1.954 | 1.107; 3.450 | **0.027** |
